# Psychometric properties of the Chinese craving beliefs questionnaire for heroin abusers in methadone treatment

**DOI:** 10.1186/1471-244X-11-39

**Published:** 2011-03-09

**Authors:** Chun-Wei Chang, Chun-Wei Huang, Wei-Hsin Wu, Bo-En Wang, Yi-Lien Liu, Hsi-Che Shen, Tony Szu-Hsien Lee

**Affiliations:** 1 Department of Psychiatry, Armed Forces Bei-Tou Hospital, Taiwan; 2 Department of Psychiatry, Lotung Poh-Ai Hospital, Taiwan; 3 Department of Psychiatry, Keelung Hospital, Department of Health, Taiwan; 4 Keelung City Health Bureau, Taiwan; 5 Public Health Bureau, Tao-Yuan County, Taiwan; 6 Taipei County Hospital and Taiwan Medical University, Taiwan; 7 Department of Health Promotion and Health Education, National Taiwan Normal University, Taiwan

## Abstract

**Background:**

This paper reports the psychometric properties of the Chinese version of Craving Beliefs Questionnaire (CCBQ), an easy-to-administer assessment instrument of measurement of craving beliefs for heroin abusers.

**Methods:**

Participants were 445 heroin abusers from four methadone clinics in Northern Taiwan. Fifty-one of the participants were tested twice within a two-week period at a different hospital to examine test-retest reliability.

**Results:**

Three-factor solution using principal component analysis was identified in the CCBQ: will power, compulsive behavior, and negative coping, accounting for 54.6% of the variance. Internal consistency analysis indicated that the three factors have strong reliability, with Cronbach alphas ranging from .81 to .92. The test-retest ICC coefficient is .80. The test-retest coefficients for the subscales will power, compulsive behavior, and negative coping are .76, .51, and .64, respectively. Overall, the data show that the CCBQ has acceptable reliability and validity, demonstrating that it can be a research instrument for assessing heroin craving beliefs.

**Conclusions:**

The psychometric properties of the CCBQ seem promising for both research and clinical purposes, and the scale thus deserves further refinement and validation with heroin abusers.

## Background

Methadone treatment is used widely as the first-choice, most available treatment for heroin dependence. Oral ingestion of methadone cannot only help opiate addicts prevent HIV infection, but it also reduces the negative consequences of heroin abuse by managing withdrawal syndromes and cravings [1,2]. Methadone maintenance therapy primarily provides heroin-dependent patients with long-term methadone and recovery plans, such as hygiene training, routine physical and mental health checkups, urine tests, and group psychological counseling [3,4]. Since the studies on the effects of methadone on HIV prevention and heroin dependence syndrome, public health researchers and psychiatrists have been investigating how best to improve the outcomes and outcome assessments further for their heroin dependent participants. A study [5] found that patients with positive perceptions of methadone on craving management showed better psychiatric functioning and stayed longer in treatment.

Methadone is a complete opioid agonist used in psychiatry for heroin detoxification [6], and so persons taking methadone should be able to delay their craving for heroin. Hence, assessing changes in heroin craving and craving beliefs during different periods of methadone consumption can provide important information regarding the efficacy of methadone treatment. A handful of instruments have been designed to assess heroin craving and craving beliefs in Western countries [7], and they have been employed either singly or in combination in pharmaceutical and/or psychosocial interventions for addiction [8,9].

Craving is a construct that continues to be actively debated in the substance dependence literature. Although there is inconsistent evidence that it is linked to relapse, craving is frequently reported as a trigger to relapse by those trying to remain abstinent and is one of the diagnostic criteria for substance dependence. Craving is viewed as a subjective experience and therefore is assessed with self-report scales. The process of craving involves physical, emotional, cognitive, and behavioral characteristics that have been well documented in the literature [10]. In addition, cravings can continue for months and even years after the cessation of drug use [11]. Rosenberg [7] provided a comprehensive review on the self report assessment tools of the craving and concluded that single-item ratings that focus on the intensity, frequency or duration of craving may fail to assess urges and craving if drug users attribute the feelings to another psychological or physiological state. Hence, single-item rating of subjective craving may not well manifest the nature of craving. Moreover, assessing different dimensions of craving may predict different types of outcome [12].

Evidence for the effectiveness of methadone programs in reducing heroin use, reducing crime, improving health, and limiting the spread of blood-borne viruses such as HIV is substantial [13]. Strategies for improving the effectiveness of existing medication programs are now an area of growing interest. Although debate about the components and diagnostic value of craving continues, reliable and valid measures of craving would have advantages for clinical psychologists, physicians, and researchers. However, few research attention has been paid to the cravings or craving beliefs of Chinese heroin dependent patients. Consequently, none of assessment tool or criteria currently available addresses the problem in Chinese culture. Hence, this paper reports the psychometric properties of the Chinese Craving Beliefs Questionnaire (CCBQ).

## Methods

### Participants and procedure

Data used in this article is from a larger longitudinal study in Taiwan aiming to assess effectiveness of methadone treatment on quality of life, mortality, craving beliefs, and HIV seroconversion. The study sample consisted of 505 heroin abusing volunteers recruited from four outpatient methadone treatment facilities located in Northern Taiwan. During the recruitment, 94 methadone patients expressed unwillingness to participate in this study. Potential participants were referred for the study by the staff of methadone treatment facilities and interviewed by trained interviewers between August and November 2008. To be included in the sample, they had to meet the DSM-IV-TR diagnostic criteria for heroin abuse [14], be at least 18 years old, and currently enrolled in a methadone program. All the participants were reimbursed NT $100 (approximately 3USD) for their participation.

In order to prevent contamination of the original study sample, the test-retest reliability of the CCBQ, additional sample of 51 methadone patients recruited from another outpatient methadone treatment program was enrolled and interviewed twice within a two-week period.

### Ethical approval

This study was approved by the Institutional Review Board of Taipei Medical University (approval number: P960205). All participants gave their written, informed consent before they completed the questionnaire.

### Measures

The CCBQ was adapted from a craving beliefs questionnaire (CBQ) developed by Wright [15], that measures beliefs about and understanding of heroin cravings. The initial version of the CCBQ consisted of 20 items translated from the English version of CBQ into Mandarin with responses given on a 7-point Likert scale ranging from totally disagree (1) to totally agree (7). The translator was fluent in both English and Mandarin, had a Ph.D. in psychology, and experience of conducting research in drug abuse for more than 10 years. The initial Mandarin version of CCBQ was then back translated by a different translator (a Ph.D. in drug offense and criminal justice). After the back-translation, the original and back-translated CCBQ were compared and all points of divergence were corrected to more accurately reflect the intent/accuracy of the item wording. The final version of CCBQ was reviewed by a group of 7 researchers in fields of psychology, psychiatry, nurse and biostatistics which determined the two language versions to be closely equivalent.

The CCBQ was then pilot tested with 7 heroin abusers in a prison to evaluate whether they can understand the CCBQ items and if the questionnaire items are related to their drug-using experience. Since the pilot test aimed to make sure that heroin abusers can cognitively understand the content and wording of items, choosing heroin abusers in the jail can give us a nice, quite space to interact and they were sober. In this initial pilot test, heroin users revised some of item wording and responded that the CCBQ format was difficult to read since they usually read Mandarin from top to down and from right to left because of traditional Mandarin classes taught in schools. They also indicated having problems with using the 7-point Likert scale and they suggested using a 5-point scale labeled 0%, 25%, 50%, 75% and 100% of agreement with the statement instead of the original 7-point scale. The translated CCBQ had 20 items in Mandarin using 5-point percentage scale oriented from top (0%) to bottom (100%) which was then used in the field-testing to evaluate its utility and selected psychometric properties.

### Statistical analysis

Five objectives were set for the development of this instrument: (1) good content and face validity; (2) short administration time; (3) simple scoring of clearly understandable items; (4) acceptable reliability; and (5) acceptable construct and concurrent validity.

The Kaiser-Meyer-Olkin (KMO) statistic was used to measure sampling adequacy and the Bartlett test of sphericity was used to determining the necessity of a factor analysis. The initial validity of the CCBQ was assessed using a principal components analysis (PCA) of a correlation matrix with Varimax rotation because we are simply transforming the original items into the new set of the principal components. The criteria for component extraction were (1) an eigenvalue ≥ 1, (2) a satisfactory result on Catell's scree test[16], (3) four or more items with salient loadings (i.e., ≥0.4), (4) reasonable internal consistency for the unit-weighted salient items (i.e., ≥0.7), and (5) adequate parsimony (mutually exclusive assignment of items to factors).

The internal consistency and test-retest reliability of the resulting components were determined by Cronbach alpha and intra-class correlation (ICC) [17]. ICCs were calculated as ratios of the variance components using a fixed-effects analysis of variance. A general-case ICC formula, described by Shrout & Fleiss [18], was adapted for the present study. An ICC above .75 indicates excellent test-retest reliability [19]. To compensate for the increased likelihood of type-1 error caused by multiple comparisons, the alpha level was adjusted so that p < .01 was required for statistical significance.

## Results

Of the initial 505 methadone treatment users in the sample, 445 completed the CCBQ satisfactorily. Twenty-eight did not complete all 20 items of CCBQ and 32 dropped of this study after completing demographics and consent forms. The demographics of 505 patients are presented in Table 1. Their mean (SD) age was 40.7 ± 9.4 years, mean age at first heroin use was 27.4 ± 7.5, and their mean (SD) education grade level was 9.18 ± 2.22. Most were men (87%); 84% had HCV; 13% were HIV positive. About 64% were employed full-time at the time of interview and 36% were unemployed. Average duration of enrolling in methadone was 184 days.

**Table 1:** Demographic and clinical characteristics according to the HIV serostatus of methadone patients from four hospitals

		Participants
		
		N = 505
Age	(years, mean ± SD)	40.7 ± 9.4
Age at first heroin use	(years, mean ± SD)	27.4 ± 7.5
Education	(years, mean ± SD)	9.18 ± 2.22
Average methadone dose at interview	(mg, mean ± SD)	48.0 ± 32.8
Duration from intake to interview	(days, mean ± SD)	184 ± 149
Gender	Male	440 (87%)
	Female	65 (13%)
Employed	No	181 (36%)
	Yes	324 (64%)
HCV	Negative	79 (16%)
	Positive	426 (84%)
HIV	Negative	439(87%)
	Positive	66 (13%)
Urine test at interview	Negative	307 (61%)
	Positive	198 (39%)

### Component structure of the CCBQ

Table 2 presents the factor loadings of the principal components analysis (PCA) and Cronbach alphas for the 3-component model. The results of the PCA for the 445 participants revealed that one CCBQ item, "When craving drugs it's OK to use alcohol to cope" has low loadings on the three factors; the loadings on factors were all less than .40 and the item-total correlation is low (r=.12) and was subsequently deleted from the CCBQ scale.

**Table 2:** The CCBQ items organized by factor

Item		Component 1	Component 2	Component 3
Component 1: will power				
16.	I cannot do anything when I am really craving heroin.	.76		
12.	The thoughts I have while craving heroin are out of my control.	.70		
20.	Craving heroin defeats my will power.	.70		
9.	I cannot stand the physical symptoms while craving heroin.	.69		
13.	The craving makes me so nervous I can't stand it.	.65		
18.	Using heroin is the only way to cope with the feeling of craving.	.63		
17.	Craving for heroin is all or none; there is nothing in between.	.53		
7.	I have no control over my behavior once the craving starts.	.52		
10.	Craving is my punishment for using heroin.	.51		
11.	If you never used heroin then you have no idea what the craving is like.	.49		
Cronbach's alpha = .88				

Component 2: compulsive behavior				
4.	4. Craving makes me use heroin.		.76	
3.	Craving drives men mad.		.73	
2.	If I don't stop, the craving will be worse.		.71	
5.	I will always have craving for heroin.		.65	
1.	The craving is a physical reaction, hence I cannot resist it.		.56	
Cronbach's alpha=.81				

Component 3: negative coping				
14.	I will never be prepared to handle the craving.			.78
8.	I will crave heroin for the rest of my life.			.74
15.	Since I will have cravings for the rest of my life, I might as well go ahead and use heroin.			.73
6.	I don't have any control over the craving.			.53
Cronbach's alpha=.81				

Variance explained by each factor		22.4%	17.4%	14.8%
Cronbach's alpha for CCBQ=.92				
Overall variance explained by the 3-factor model: 54.6%				

The results indicated that the remaining 19 items fit a 3-component solution that meets the criteria listed in Methods (eigenvalue ≥ 1; factor loading ≥ .40; Alpha ≥ .70). These three factors together explain 54.6% of the total variance and yield excellent internal consistency coefficients (≥0.8). The first and most influential component consists of 10 items and explains 22.4% of the total variance; this component was labeled "will power." The second component includes 5 items and explains 17.4% of the total variance; this component was labeled "compulsive behavior." The third component retains four items and explains 14.8% of the total variance; this component was labeled "negative coping." The items comprising these components are listed in Table 2. The sampling adequacy of the model is good (KMO = 0.93) and the Bartlett test of sphericity is significant (445, p < .01), indicating that the model fit is good.

### Reliability: Internal-consistency and test-retest

Of the 51 participants enrolled in test-retest phase of the study, 46 filled out the CCBQ again after two weeks and 5 lost of contact. The results show that the test-retest reliability using the ICC coefficient is .80. The test-retest coefficients for the components "will power," "compulsive behavior," and "negative coping" are .76, .51, and .64, respectively.

Possible CCBQ scores then range from 19 to 95 (*M *= 59.9, *SD *= 13.1). The CCBQ demonstrated acceptable internal consistency, with Cronbach alpha for the total scale equaling .92; the alphas for the three components range from .81 to .88 (Table 2).

## Discussion

The final version of the CCBQ contains 19 items rated on a 5-point percent agreement scale. The results of principal components analysis of data collected from heroin abusing individuals in Taiwan using the CCBQ correspond closely with the results of previous research representing heroin craving as related to three domains: will power [20], compulsive behavior [21,22], and negative coping [23]. One item about using alcohol to cope craving of heroin had to be deleted from the scale due to low factor loading and low item-total correlation. We speculate that is because when alcohol is a surrogate for heroin, social adjustment improves, but the medical outcome is worsened [24]. Overall, the results of our study are also consistent with Tiffany's proposition that craving is conceptually multi-dimensional [25].

A central objective in developing the CCBQ was to create a short and easy to administer assessment instrument with good psychometric properties. For most participants, administration of the CCBQ took an average of 10 minutes. The items were also designed to be easy to score and understand. The factor loadings, item correlations, and Cronbach alpha values are similar to those reported for other rating scales [26]. In sum, this brief scale is composed of clear, simple items requiring little judgment for scoring, and it meets generally accepted psychometric standards.

In conclusion, the results of the study indicate that the reliability and validity of the CCBQ are promising and that the scale deserves further refinement and more validation for use with drug abusers. The evidence also supports the conclusion that the collection of reliable data by research or treatment personnel on the problems experienced by heroin abusers for outcome evaluation purposes need not be time-consuming. The results also have several practical implications for outcome research. If the CCBQ is to be used in routine clinical practice, it may be advantageous to incorporate it within existing assessment protocols. The short amount of time required to administer the CCBQ is encouraging in this regard. In short, the overall findings of the study support the efficacy and reliability of the CCBQ and its potential for further use for both research and clinical purposes.

Finally, a few limitations of the study should be mentioned. The participants were recruited from outpatient methadone treatment clinics and our sample was not randomly sampled. In addition, although the sample was large, relatively diverse, and fairly typical of the demographic makeup of heroin abusers in Taiwan, the fact remains that it was restricted to Chinese patients living in Taiwan. Since the alcohol abuse was not assessed, the lack of comorbidity assessment should be highlighted as a limitation of the study. Therefore, caution should be exercised in applying the proposed CCBQ structure to other ethnic groups and in other countries. Nonetheless, the sample used for the study and the attendant analyses of the CCBQ data should provide a good launching point for future tests of the construct, concurrent, and convergent validity of the CCBQ and for treatment-outcome research generally.

## Conclusions

The primary findings in this article support that the 19-item CCBQ meets the criteria of a short and easy to administer assessment instrument with great psychometric properties. The findings of the CCBQ seem promising for both heroin craving related research and clinical purposes.

## Competing interests

The authors declare that they have no competing interests.

## Authors' contributions

CWC carried out analysis and draft the manuscript. CWH, WHW, BEW, and YLL participated in data collection, results interpretation and discussion of this manuscript. HCS participated in critical review of introduction and discussion. TSHL conceived, planned and conducted the study. TSHL also draft and revised the manuscript. All authors read and approved the final manuscript.

## Pre-publication history

The pre-publication history for this paper can be accessed here:

http://www.biomedcentral.com/1471-244X/11/39/prepub
